# The implementation of malaria intermittent preventive trialtreatment with sulphadoxine-pyrimethamine in infants reduced all-cause mortality in the district of Kolokani, Mali: results from a cluster randomized control

**DOI:** 10.1186/1475-2875-11-73

**Published:** 2012-03-16

**Authors:** Alassane Dicko, Moussa Konare, Djibril Traore, Jean Testa, Roger Salamon, Ogobara Doumbo, Christophe Rogier

**Affiliations:** 1 Malaria Research and Training Center, Faculty of Medicine Pharmacy and Dentistry, University of Bamako, P.O. Box 1805, Bamako, Mali; 2 Department of Public Health, Faculty of Medicine Pharmacy and Dentistry, University of Bamako, P.O. Box 1805, Bamako, Mali; 3 Institut de Santé Publique, d'Épidémiologie et de Développement, Université Victor Segalen Bordeaux 2, Case 11 146 Rue Léo Saignat, Bordeaux Cedex 33076, France; 4 Institut de Recherche Biomédicale des Armées IRBA-ex-IMTSSA & UMR6236-URMITE, Allée du Médecin colonel Jamot, Parc du Pharo, BP60109, 13262 Marseille cedex 07, France; 5 Institut Pasteur de Madagascar, B.P. 1274, 101, Antananarivo, Madagascar

**Keywords:** Mortality, Malaria, Intermittent preventive treatment, Sulphadoxine-pyrimethamine, Expanded programme of immunization

## Abstract

**Background:**

Malaria intermittent preventive treatment of malaria in infant with sulphadoxine-pyrimethamine (IPTi-SP) reduced the incidence of malaria and anaemia by 30% and 20% respectively. The strategy is now a recommended policy for malaria control. However, there was no published study on the impact of the strategy on mortality. The present study assessed the impact of the implementation of IPTi-SP in health services in Mali on all-cause mortality.

**Methods:**

The 22 health sub-districts of the district of Kolokani were randomized at a 1:1 ratio to either receive IPTi-SP or to serve as a control. The IPTi-SP was implemented for two years starting December 2006. Information on births and deaths through 31 March, 2009 was collected on all children who reached four months of age on 1 December, 2006, likely to be exposed to the intervention in 75 localities randomly selected in each zone.

**Results:**

A total of 5,882 children (2,869 from the intervention zone and 3,013 from the nonintervention zone) who reached four months of age between 1 December, 2006 and 1 December, 2008 were surveyed between the age of four months to the age of 18 months from 1 December, 2006 to 31 March, 2009. In the cohort of four to 18 months of age, the mortality rate per 1,000 children was 2.53 in the intervention zone compared to 3.46 in the nonintervention zone, gender and season adjusted mortality rate ratio (MRR) = 0.73 (95% CI 0.55-0.97, p = 0.029). In the cohort of the four to 12 months of age, mortality rates per 1,000 children were 2.22 in the intervention zone and 3.13 in the non-intervention zone, MRR = 0.71 (95% CI 0.49-1.02, p = 0.064) adjusted for gender and season.

**Conclusion:**

The implementation of the IPTi-SP resulted in a substantial reduction in all-cause mortality in children. The results of this study support the adoption and the implementation of IPTi-SP as malaria control strategy.

**Trial Registration:**

ClinicalTrials.govNCT00766662

## Background

It is estimated that about one million of people in sub-Saharan Africa died directly from malaria in 2008, of which 800,000 were children under five years [1]. The numbers are higher if indirect contribution of malaria is taken into account [2,3]. In the absence of licensed vaccine against malaria, intermittent preventive treatment of malaria in infants (IPTi) consisting of the administration of a curative dose of sulphadoxine-pyrimethamine (SP) at the time of EPI vaccination (DTP2, DTP3 and measles vaccine) regardless of the presence of symptoms or infection was developed to reduce the burden of malaria. Randomized control studies have shown that the strategy reduced the incidence of malaria by 30% and the incidence of anaemia by 20% [4]. The strategy is now recommended as policy for malaria control in infants in areas of moderate or high malaria transmission and where the resistance to SP is low [5]. However, there was no published study on the impact of the strategy on the mortality. The sample size and the duration of follow-up of initial studies, which assessed the efficacy of the strategy on the clinical episodes, did not allow the assessment of its efficacy on mortality. The pilot implementation of IPTi in health services in Mali offered an opportunity to address this question. The objective of the present study was to assess the impact of IPTi implementation in health services in Mali on the mortality in the targeted population.

## Methods

### Study site

The study was conducted in the district of Kolokani, Mali. The district covers 14,380 km^2^, divided into 22 health sub-districts. Each health sub-district covers several villages (average of 13 villages/health sub-district). The total population was 208,317 inhabitants with children under one year of age representing about 4% of the total population. Malaria is hyperendemic in the region with parasite prevalence in children under five years of 45% during the dry season and above 70% during the rainy season [6]. Each sub-district was managed by a physician or a nurse assisted by other nurses. The district hospital located in Kolokani is the reference centre for the 22 heath sub-districts. A team composed of physicians, midwives, nurses and managers ensure the activities at the district heath centre and the coordination and supervision of the activities in the health sub-districts.

### Study participants and design

The study was a cluster randomized trial. The 22 health sub-districts were randomized in a 1:1 ratio with the intervention in 11 health areas and the other 11 serving as controls for the assessment of the impact of IPTi implementation on the mortality. The intervention consisted of the administration of a half tablet of SP along with EPI vaccines: second and third dose of the DTP at the age of three and four months respectively and measles and yellow fever vaccines at nine months of age. The implementation started in December 2006 after the training of the health personnel and provision of SP to the health centres. This implementation was co-supervised by the Malaria Research and Training Center (MRTC) of the University of Bamako and the district health services through December 2007. The implementation continued in the intervention zones through December 2008 under the supervision of the health services. Sulphadoxine-pyrimethamine used during the second year was provided by UNICEF to the district health services. A cross-sectional survey conducted after one year of the implementation in December 2007 found that 77% of children who received the EPI vaccines also received the IPTi-SP in the intervention zone while this proportion was less than 3% in the non-intervention zone [7]. The impact of the implementation on all-cause mortality was assessed in April 2009 using a cross-sectional survey. UNICEF was not involved with this evaluation of the effect on mortality.

### Sample size and sampling

Using an approximation of arc-sinus transform formula, assuming an expected mortality rate of 106 for 1,000 person-months in children aged four to 18 months, to detect a 30% difference in mortality between the two zones with an alpha error of 5% and a power of 80%, about 1,263 subjects in each zone were needed if a simple random sampling method was used. Assuming a cluster effect of 1.5, taking into account the cluster sampling and a proportion of missing data of 5%, a total of 1,995 subjects per zone were needed. With a total population estimated at 214,509 inhabitants and a proportion of children aged four to 18 months of 4% (8,580) about half the total population (i.e. half the localities in the district) was to be surveyed.

A total of 150 localities were randomly selected and each household was visited. A simple questionnaire was designed and used collect information in all the children, who reached age of four months between 1 December, 2006 and 1 December, 2008 (born between 1 August, 2006 and 1 August, 2008) likely to be exposed to the intervention between 1 December, 2006 and 31 December, 2008. Data were collected by interview and using the child's birth certificate, vaccination card or mother antenatal consultation records. Information collected include the locality, the sub-district, the compound, the household number and subject number in the household, date of birth, vital status (died or not) and date of death if applicable.

### Data management and analysis

Data were collected on forms, were transferred in Bamako and double entered using Microsoft Access and reconciled. Statistical analysis was performed using Stata, version 10 (Houston Texas, USA). The primary endpoint was the all-cause mortality between 1 December, 2006 and 31 March, 2009 in children aged four to18 months (cohort 1). The secondary endpoint was the all-cause death rate between 1 December, 2006 and 31 March, 2009 in children aged four to 12 months (cohort 2). Cohort 2 was included in cohort 1. The death rates in the two zones were compared using Fischer exact test. Cox regression model adjusted for gender and stratified by trimester was used to adjust for the potential effect of the transmission season and gender. The starting point was the date of age of four months (to start the exposition to the intervention) and the ending point was either: i) the date of age of 18 months or 12 months for cohort 1 and cohort 2 respectively, or ii) date of death, or iii) 31 March, 2009. The duration of follow-up (period at risk) for each subject was the number of days between the starting and the ending points.

### Ethical considerations

Initial and successive amendments of the protocol were examined and approved by the Ethical Committee of the Faculty of Medicine, Pharmacy and Dentistry of the University of Bamako. Informed consents were obtained from parents prior to interview.

## Results

A total of 5,882 children that reached four months of age between 1 December, 2006 and 1 December, 2008 in the study area were surveyed, including 2,869 from 75 localities in the intervention zone and 3,013 from 75 villages in the non-intervention zone. The gender distribution was similar between the two zones (50.5% in the intervention zone and 51.6% in the non-intervention zone were male, p = 0.43). The exact date of death was unknown for two subjects (a boy in the intervention zone born on 15 February, 2007 and a girl born on 10 January 2008, in the non-intervention zone) who were excluded from the analysis.

**Figure 1:**
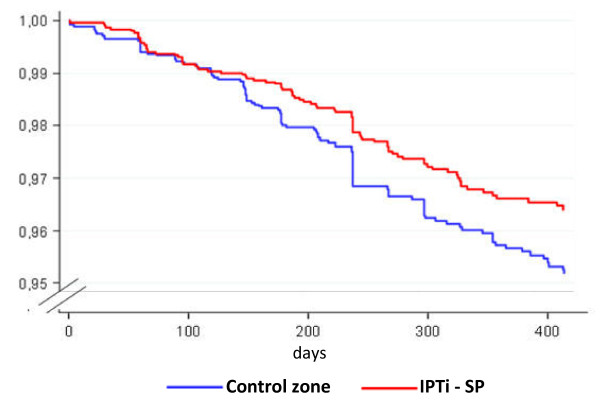
**Kaplan Meir survival estimates of time to death from age of four months in the two zones**.

Among the 5,880 children, 198 died at the age of four to 18 months and 119 died at the age of four to 12 months. Of the children who died at age of four to 18 months, 86 (2.86%) lived in the intervention and 112 (3.91%) lived in the non-intervention zone (Fischer exact test; two sided p-value p = 0.030). Of those who died at the age of four to 12 months, 51 (1.69%) lived in the intervention zone and 68 (2.37%) lived in the non-intervention zone (Fischer exact test; two sided p-value p = 0.078). The mortality rate, mortality rates ratios and reduction in mortality are presented in Table 1. In the cohort of four to 18 months of age, the mortality rate per 1,000 children was 2.532 in the intervention zone compared to 3.456 in the nonintervention zone, gender-adjusted mortality rates ratio (MRR) = 0.73 (95% CI 0.55-0.97, p = 0.029). In the cohort of the four to 12 months of age, mortality rates per 1,000 children were 2.223 in the intervention zone and 3.126 in the non-intervention zone, adjusted for gender of MRR = 0.71 (95% CI 0.49-1.02, p = 0.64). The curves of the Kaplan-Meier survival estimates of time to death in children from four months shows significant difference in survival between the two zones (p <0.001) (Figure 1)

**Table 1:** Mortality rates, mortality rates ratios and reductions in mortality in the two cohorts in the intervention and non-intervention zone

	Cohort 1 (4-18 months of age)			Cohort 2 (4-12 months of age)	
	**IPTi-SP**	**Control**		**IPTi-SP**	**Control**

Death	86	112		51	68

Person-days	1,018,825	971,905		688,294	652,688

Mortality rate per 1000 children	2.532	3.456		2.223	3.126

Unadjusted RR (95% IC)	0.73 (0.55-0.98)			0.71 (0.48-1.04)	

p	0.030			0.066	

Adjusted* RR (IC 95%) in Cox model	0.73 (0.55-0.97)			0.71 (0.49-1.02)	

p* (two-sided)	0.029			0.064	

Reduction in mortality* (%)	27 (3-45)			29 (0-49)	

* adjusted for gender in Cox model

## Discussion

This study estimated the effect of two years of IPTi implementation on mortality in children and has found a reduction in all-cause mortality by 27% and 29% in children four to 18 months of age and those of four to 12 months of age, respectively. Use of insecticide-impregnated bet nets (ITN) for malaria control in endemic areas was associated with a reduction of 17% in all-cause mortality [8]. The implantation of IPTi along with EPI resulted in a reduction in all-cause mortality greater than the reduction provided by ITN, a current recommended strategy for malaria control.

Demonstrating the effect of a malaria control strategy on malaria specific mortality has always been difficult due to the requirement of large sample size and factors that can confound the relationship between the intervention and the malaria-specific mortality. For example, the impact of the use of the ITN on malaria-specific mortality could not be demonstrated initially in individual, large, randomized control trials [9,10], but only in the meta-analysis studies [8,11]. Attributing a death to malaria is difficult as the current indirect methods, such as verbal autopsies, lack sensitivity and specificity [12]. The effect of a malaria intervention on all-cause mortality was reported in previous studies [9,13,19]. Although, it requires a larger number of subjects than malaria-specific mortality assessment, there is no issue of sensitivity or the specificity when assessing it. For these reasons, the objective of the present study was limited to the assessment of the impact of the IPTi implementation on all-cause mortality. Additional reason for focusing on this objective was the fact that the data were collected retrospectively and far from the events, which made it difficult to accurately assign the cause of the death (including when verbal autopsy methods were used).

In the present study, the reduction in all-cause mortality may be due to a combination of both the direct and indirect effect of the IPTi on malaria and the indirect effects on other causes of death. Previous evaluations have shown that the implementation of IPTi has resulted in a significant increase in coverage of EPI vaccines, an increase that was more marked in the intervention zone [7]. The higher increase in vaccine coverage in the intervention zone may have contributed to the increase in child survival in these areas. Previous studies have shown that Bacille Calmette-Guerin (BCG) vaccination and measles vaccination have strong beneficial effect on child survival [14-16]. It is also known that a substantial part of the mortality due to malaria is not directly attributable to malaria (i.e., indirectly attributed to malaria) [17] and results of adequately executed malaria control or elimination programmes could exceed expectations due to decreased indirect malaria mortality [18-20]. Malaria infections may alter the capacity to survive other affections for example through chronic anaemia and enhancement of the severity of other childhood diseases.

IPTi has now been adopted by the WHO as a policy for malaria control despite the lack of data on the impact of the strategy on mortality [5,21,22].

This study has two major strengths. First, it is a randomized controlled study, which ensures the comparability between the two arms. Second, the IPTi was implemented within the health care system, better reflecting the environment in which the strategy will be implemented. Although the data were collected by individuals not involved in the implementation and who were not aware if a locality was in the intervention or non-intervention zone, it is difficult to rule out the possibility of observer bias, since it would be easy to find out if a locality was in the intervention or non intervention zone. The mortality rate observed in the present study was lower than expected (40.8 per 1,000 in the non-intervention zone compared to 106 per 1,000 at the national level according to the national health survey) resulting in relatively lower power and wider confidence intervals. Reassuringly, the mortality rates in this study are consistent with those reported elsewhere in the West African region [14,23,24]. Another limitation of the study is the relatively shorter period of the intervention (two years). It is possible that the impact of the strategy on the mortality increases with the duration of the intervention (for example, reduction in the malaria parasite carriage leading to a reduction in the transmission). It is therefore important that the impact of the implementation of the strategy continue to be monitored over longer periods of time during the implementation of the strategy as policy.

## Conclusion

The implementation of the IPTi has resulted in a 27% reduction of the all-cause mortality in children aged four to 18 months. The results of this study support the adoption of IPTi as malaria control strategy and its implementation as a tool for malaria control.

## Competing interests

The authors declare that they have no competing interests.

## Authors' contributions

AD, OD, JT, RS, CR conceived and designed the study. AD, OD, MK, DT planned, organized, supervised the collected data. Data were analysed by AD and CR. All authors read and approved the final manuscript
